# Comparative Genome Analysis of Extended-Spectrum-β-Lactamase-Producing *Escherichia coli* Sequence Type 131 Strains from Nepal and Japan

**DOI:** 10.1128/mSphere.00289-16

**Published:** 2016-10-26

**Authors:** Tohru Miyoshi-Akiyama, Jatan Bahadur Sherchan, Yohei Doi, Maki Nagamatsu, Jeevan B. Sherchand, Sarmila Tandukar, Norio Ohmagari, Teruo Kirikae, Hiroshi Ohara, Kayoko Hayakawa

**Affiliations:** a Pathogenic Microbe Laboratory, Research Institute, National Center for Global Health and Medicine, Tokyo, Japan; b Department of Clinical Microbiology, Kathmandu University School of Medical Sciences, Dhulikhel, Nepal; c Division of Infectious Diseases, University of Pittsburgh School of Medicine, Pittsburgh, Pennsylvania, USA; d Disease Control and Prevention Center, National Center for Global Health and Medicine, Tokyo, Japan; e Department of Infectious Diseases, Research Institute, National Center for Global Health and Medicine, Tokyo, Japan; f Public Health Research Laboratory, Institute of Medicine, Tribhuvan University Teaching Hospital, Kathmandu, Nepal; g Department of International Medical Cooperation, National Center for Global Health and Medicine, Tokyo, Japan; Antimicrobial Development Specialists, LLC

**Keywords:** CTX-M, *Escherichia coli*, ST131, antimicrobial resistance, extended-spectrum beta-lactamase, whole-genome sequence

## Abstract

The global spread of extended-spectrum-β-lactamase (ESBL)-producing *Escherichia coli* (ESBL-*E. coli*) has largely been driven by the pandemic sequence type 131 (ST131). This study aimed to determine the molecular epidemiology of their spread in two Asian countries with contrasting prevalence. We conducted whole-genome sequencing (WGS) of ESBL-*E. coli* ST131 strains collected prospectively from Nepal and Japan, two countries in Asia with a high and low prevalence of ESBL-*E. coli*, respectively. We also systematically compared these genomes with those reported from other regions using publicly available WGS data for *E. coli* ST131 strains. Further, we conducted phylogenetic analysis of these isolates and all genome sequence data for ST131 strains to determine sequence diversity. One hundred five unique ESBL-*E. coli* isolates from Nepal (February 2013 to July 2013) and 76 isolates from Japan (October 2013 to September 2014) were included. Of these isolates, 54 (51%) isolates from Nepal and 11 (14%) isolates from Japan were identified as ST131 by WGS. Phylogenetic analysis based on WGS suggested that the majority of ESBL-*E. coli* ST131 isolates from Nepal clustered together, whereas those from Japan were more diverse. Half of the ESBL-*E. coli* ST131 isolates from Japan belonged to virotype C, whereas half of the isolates from Nepal belonged to a virotype other than virotype A, B, C, D, or E (A/B/C/D/E). The dominant sublineage of *E. coli* ST131 was *H*30Rx, which was most prominent in ESBL-*E. coli* ST131 isolates from Nepal. Our results revealed distinct phylogenetic characteristics of ESBL-*E. coli* ST131 spread in the two geographical areas of Asia, indicating the involvement of multiple factors in its local spread in each region.

**IMPORTANCE** The global spread of ESBL-*E. coli* has been driven in large part by pandemic sequence type 131 (ST131). A recent study suggested that, within *E. coli* ST131, certain sublineages have disseminated worldwide with little association with their geographical origin, highlighting the complexity of the epidemiology of this pandemic clone. ST131 bacteria have also been classified into four virotypes based on the distribution of certain virulence genes. Information on virotype distribution in Asian ST131 strains is limited. We conducted whole-genome sequencing of ESBL-*E. coli* ST131 strains collected in Nepal and Japan, two Asian countries with a high and low prevalence of ESBL-*E. coli*, respectively. We systematically compared these ST131 genomes with those reported from other regions to gain insights into the molecular epidemiology of their spread and found the distinct phylogenetic characteristics of the spread of ESBL-*E. coli* ST131 in these two geographical areas of Asia.

## Abstract (precis)

The global spread of ESBL-*E. coli* has been driven in large part by pandemic sequence type 131 (ST131). A recent study suggested that, within *E. coli* ST131, certain sublineages have disseminated worldwide with little association with their geographical origin, highlighting the complexity of the epidemiology of this pandemic clone. ST131 bacteria have also been classified into four virotypes based on the distribution of certain virulence genes. Information on virotype distribution in Asian ST131 strains is limited. We conducted whole-genome sequencing of ESBL-*E. coli* ST131 strains collected in Nepal and Japan, two Asian countries with a high and low prevalence of ESBL-*E. coli*, respectively. We systematically compared these ST131 genomes with those reported from other regions to gain insights into the molecular epidemiology of their spread and found the distinct phylogenetic characteristics of the spread of ESBL-*E. coli* ST131 in these two geographical areas of Asia.

## INTRODUCTION

The emergence of extended-spectrum-β-lactamase (ESBL)-producing *Escherichia coli* (ESBL-*E. coli*) is a global problem. However, its prevalence and epidemiology differ significantly depending on its geographical location. According to a recent report from the World Health Organization (WHO) (1), the prevalences of *E. coli* resistance to third-generation cephalosporins were 14% in Europe, 27% in the Americas (15% in the United States), and 30% in Southeast Asia. The prevalence of *E. coli* resistance to these cephalosporins was the highest in developing countries in Asia (38% in Nepal, 68% in Myanmar, and 16% to 95% in India) while relatively low in more-developed Asian countries, including Japan (17%) (1).

The global spread of ESBL-*E. coli* has been driven in large part by pandemic sequence type 131 (ST131); *E. coli* ST131 bacteria are often resistant to multiple drugs (2). A recent study suggested that, within *E. coli* ST131, certain sublineages have disseminated worldwide with little association with their geographical origin, highlighting the complexity of the epidemiology of this pandemic clone (3). ST131 has also been classified into four virotypes based on the distribution of certain virulence genes, and virotype C has been reported to be globally disseminated (4). However, information pertaining to virotype distribution among Asian ST131 strains is limited.

In this study, we conducted whole-genome sequencing (WGS) of ESBL-*E. coli* ST131 strains collected prospectively in Nepal (5) and Japan. Although both countries are located in Asia, the socioeconomic statuses of Japan and Nepal are different and they also have distinct prevalences of ESBL-*E. coli* (Japan has a low prevalence, and Nepal has a high prevalence). We systematically compared these ST131 genomes with those reported from other regions to gain insights into the epidemiology of their spread.

## RESULTS AND DISCUSSION

### Characteristics of ESBL-*E*. *coli* ST131 isolates and *E. coli* ST131 genomes.

During the study period, 105 and 76 unique ESBL-*E. coli* isolates were identified from Nepal and Japan, respectively. Of these isolates, 54 (51%) isolates from Nepal and 11 (14%) isolates from Japan were identified as ST131 by WGS based on sequence typing and included in further analyses. Forty (38.1%) of 105 ESBL-*E. coli* isolates from Nepal and 19 (25%) of 76 ESBL-*E. coli* isolates from Japan were isolated from outpatients. Nineteen (35.2%) of 54 ESBL-*E. coli* ST131 isolates from Nepal and 2 (18.2%) of 11 of ESBL-*E. coli* ST131 isolates from Japan were from outpatients. This suggests that ESBL-*E. coli* ST131 bacteria were isolated from outpatients almost twice as often in Nepal as in Japan.

The median age of patients with ESBL-*E. coli* ST131 was significantly lower in Nepal (39 years [interquartile range {IQR}, 25 to 61 years]) than in Japan (83 years [IQR, 73 to 86 years]) (*P* < 0.001). The numbers of male patients with ESBL-*E. coli* ST131 were similar in the two countries (19 [35%] in Nepal and 4 in Japan [36%]). The majority of isolates were collected from urine samples (54 [100%] in Nepal and 8 [73%] in Japan). Pregnant female patients with ESBL-*E. coli* ST131 were more frequent in Nepal (*n* = 9 [26%]) than in Japan (*n* = 0) (*P* < 0.001). Comorbid conditions, such as malignancy (*n* = 2 [3.7%] in Nepal; *n* = 3 [27.3%] in Japan) and underlying urological conditions (e.g., benign prostatic hyperplasia, urolithiasis, obstructive urinary diseases) (*n* = 10 [18.5%] in Nepal; *n* = 6 [54.5%] in Japan) were more common in patients with ESBL-*E. coli* in Japan than in patients in Nepal (*P* = 0.031 and *P* = 0.02, respectively). ESBL-*E. coli* isolates from Nepal and Japan had distinct clinical characteristics.

In a study that identified ESBL-*E. coli* from travelers who returned from India, 47% of 17 ESBL-*E. coli* isolates collected from 2004 to 2006 were ST131 (6). The prevalence of ST131 among ESBL-*E. coli* bacteria isolated in Nepal in our study is in line with the previous data, considering the geographical proximity and traffic of visitors between Nepal and India. The ST131 prevalence of 14% found among ESBL-*E. coli* isolates from Japan is also similar to that reported in a previous study where 21% of 130 ESBL-*E. coli* isolates collected between 2002 and 2003 from Japan were ST131 (7). However, the prevalence was lower than that reported in a more recent study conducted in Japan, in which 37% of 581 ESBL-*E. coli* isolates collected from 2001 to 2010 were identified as ST131 (8). This discrepancy might be attributed to the differences in the year of collection, geographical locations in the country, and methods used to identify ST131 (PCR-based screening versus WGS).

### *fimH* alleles and *H*30Rx sublineages in *E. coli* ST131.

*fimH* alleles and *H*30Rx sublineages in *E. coli* ST131 were determined and compared among various geographical regions (Table 1). *fimH30*, especially *fimH30*Rx comprised the majority of the *fimH* alleles in various geographical regions, followed by *fimH41* and *fimH22*. *fimH27* was observed only in *E. coli* ST131 from Africa. We found that all 54 ESBL-*E. coli* isolates from Nepal belonged to the *H*30Rx sublineage. There is little data on the prevalence of the *H*30Rx sublineage in South Asia. A previous study that included only a few ST131 strains from India revealed that all of them belonged to clade C (i.e., *H*30) and produced CTX-M-15 (3). Our result is in accordance with the high prevalence of CTX-M-15-producing ESBL-*E. coli* reported among travelers returning from the Indian subcontinent (6). However, the limited isolate collection period in one facility might have affected the clonal distribution of ESBL-*E. coli* ST131 isolates from Nepal in our study. *H*30R (non-Rx) were more prevalent among ESBL-*E. coli* ST131 isolates from Japan compared to isolates from Nepal, although more than 60% of ESBL-*E. coli* ST131 isolates from Japan still belonged to *H*30Rx. A previous report from Japan suggested a higher prevalence of *H*30R (non-Rx) and *H*22 among ESBL-*E. coli* ST131 isolates collected from 2001 to 2012 in 10 Japanese acute-care hospitals located in Kyoto, Shiga area, which is more than 460 km (286 miles) away from the hospital in this study (9). The relatively high prevalence of *H*30R (non-Rx) and *H*22 observed in this study in the United States and Europe, respectively, were consistent with previous reports (3, 10).

**TABLE 1:** Comparison of *fimH* alleles and *H*30Rx and *H*30R sublineages in *Escherichia coli* ST131

*fimH* allele or sublineage	Total no. of isolates (*n* = 132)	No. of isolates (%) from:						*P* value^d^
		Nepal (*n* = 54)	Japan (*n* = 12)^a^	Other Asian countries (*n* = 3)^b^	USA (*n* = 29)^b^	Europe (*n* = 29)^b^^,^^c^	Africa (*n* = 5)^b^	
*H*30	110	54 (100)	11 (91.7)	3 (100)	27 (93.1)	12 (41.4)	3 (60)	**<0.001**
*H*30Rx	88	54 (100)	8 (66.7)	2 (66.7)	16 (55.2)	6 (20.7)	2 (40)	**<0.001**
*H*30R (non-Rx)	19	0 (0)	3 (25)	1 (33.3)	11 (37.9)	3 (10.3)	1 (20)	**0.006**
*H*22	9	0 (0)	0 (0)	0 (0)	0 (0)	9 (31)	0 (0)	**<0.001**
*H*27	2	0 (0)	0 (0)	0 (0)	0 (0)	0 (0)	2 (40)	**<0.001**
*H*41	11	0 (0)	1 (8.3)	0 (0)	2 (6.9)	8 (27.6)	0 (0)	**0.001**

a Includes one publicly available sequence of *E. coli* ST131.

b Publicly available sequence data for *E. coli* ST131.

c One isolate from Austria belonged to *H*31.

d The *P* values are comparing the prevalence of each antibiotic resistance gene among geographical regions. *P* values in boldface type represent statistically significant results.

### Antimicrobial susceptibility and antibiotic resistance genes.

**FIGURE 1:**
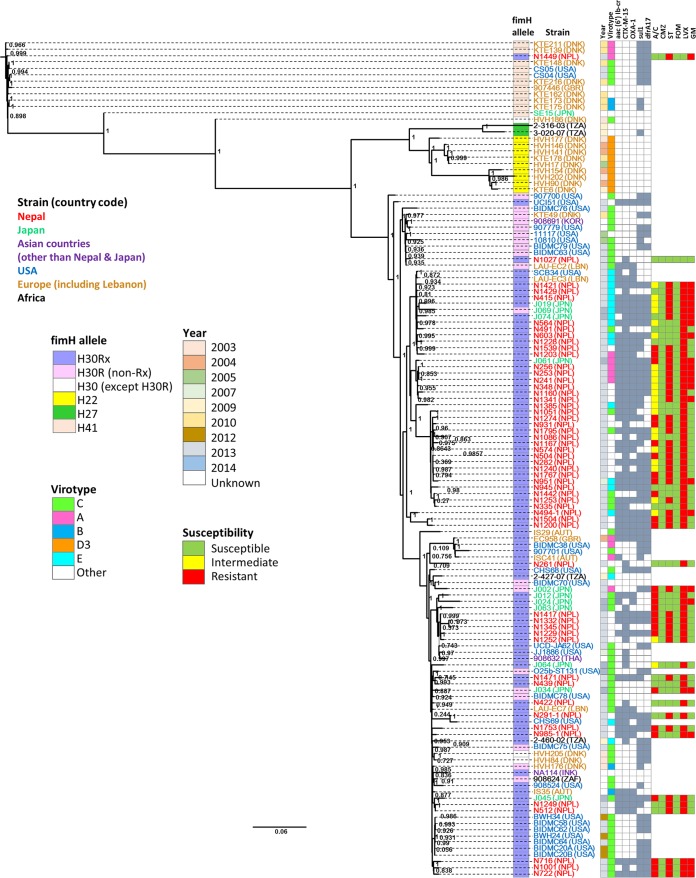
Phylogenetic trees of ST131 strains. ML phylogenetic trees were estimated using PHYML 3.0 and SH statistics for branch support. Antibiotics are abbreviated as follows: A/C, amoxicillin and clavulanate; CMZ, cefmetazole; ST, trimethoprim-sulfamethoxazole; FOM, fosfomycin; LVX, levofloxacin; GM, gentamicin. The countries are shown in parentheses after the strain name and abbreviated as follows: DNK, Denmark; NPL, Nepal; GBR, Great Britain; JPN, Japan; TZA, Tanzania; KOR, South Korea; LBN, Lebanon; AUT, Austria; THA, Thailand. Susceptibility data are available only for the 11 ESBL-*E. coli* ST131 isolates from the National Center for Global Health and Medicine, Japan, and 54 ESBL-*E. coli* ST131 isolates from Tribhuvan University, Nepal. For isolates from Japan, ampicillin-sulbactam was used instead of amoxicillin-clavulanate. For resistance genes, a filled square means positive for the resistance gene and an open square means negative for the resistance gene.

Antimicrobial susceptibility data were available for 54 and 11 ESBL-*E. coli* ST131 isolates from Nepal and Japan, respectively (Fig. 1). The susceptibilities of the 105 ESBL-*E. coli* isolates (including 54 ESBL-*E. coli* ST131 isolates described in this study) from Nepal were described elsewhere (5). The susceptibilities to trimethoprim-sulfamethoxazole, gentamicin, and levofloxacin were not significantly different in isolates from Nepal and Japan. All ESBL-*E. coli* ST131 isolates from Nepal and Japan were susceptible to cefmetazole and fosfomycin.

The frequency of resistance genes among the *E. coli* ST131 isolates is summarized in Table 2. More than 94% (*n* = 51) and 66% (*n* = 8) of ESBL-*E. coli* ST131 isolates were positive for *bla*_CTX-M-15_ in Nepal and Japan, respectively. Overall, 50 (70%) of the 71 *bla*_CTX-M-15_-positive isolates in the entire cohort were also positive for *bla*_OXA-1_ and *aac*(*6′*)-*Ib*-*cr* (encoding an aminoglycoside/fluoroquinolone acetyltransferase); of these 71 isolates, 38 isolates were obtained from Nepal, 5 isolates from Japan, and 6 isolates from Europe.

**TABLE 2:** Comparison of resistance genes in *Escherichia coli* ST131

Antibiotic(s) and antibiotic resistance gene	*fimH* allele or sublineage	Total no. of isolates (*n* = 132)	No. of isolates (%) from:						*P* value^d^
			Nepal (*n* = 54)	Japan (*n* = 12)^a^	Other Asian countries (*n* = 3)^b^	USA (*n* = 29)^b^	Europe (*n* = 29)^b^^,^^c^	Africa (*n* = 5)^b^	
Aminoglycoside									
*aac*(*3*)-*IIa*	Total	26	15 (27.8)	5 (41.7)	0	3 (10.3)	3 (10.3)	0	0.05
	*H*30Rx	25	15 (27.8)	4 (33.3)		3 (10.3)	3 (10.3)		
	*H*30R	1		1 (8.3)					
*aac*(*3*)-*IId*	Total	17	1 (1.9)	2 (16.7)	1 (33.3)	5 (17.2)	5 (17.2)	3 (60)	**0.003**
	*H*30Rx	3	1 (1.9)			1 (3.4)		1 (20)	
	*H*30R	6		2 (16.7)	1 (33.3)	2 (6.9)		1 (20)	
	*H*27	1						1 (20)	
	*H*41	7				2 (6.9)	5 (17.2)		
*aadA1*	Total	5	0	0	0	2 (6.9)	3 (10.3)	0	0.213
	*H*30Rx	1					1 (3.4)		
	*H*30R	3				2 (6.9)	1 (3.4)		
	*H*41	1					1 (3.4)		
*aadA2*	Total	11	8 (14.8)	0	0	2 (6.9)	1 (3.4)	0	0.329
	*H*30Rx	8	8 (14.8)						
	*H*30R	3				2 (6.9)	1 (3.4)		
*aadA5*	Total	68	31 (57.4)	6 (50)	0	21 (72.4)	9 (31)	1 (20)	**0.007**
	*H*30Rx	51	31 (57.4)	4 (33.3)		13 (44.8)	2 (6.9)	1 (20)	
	*H*30R	9		2 (16.7)		6 (20.7)	1 (3.4)		
	*H*41	8				2 (6.9)	4 (13.8)		
*strA*	Total	45	20 (37)	2 (16.7)	1 (33.3)	13 (44.8)	6 (20.7)	3 (60)	0.211
	*H*30Rx	28	20 (37)	1 (8.3)		6 (20.7)		1 (20)	
	*H*30R	7		1 (8.3)	1 (33.3)	5 (17.2)			
	*H*27	2						2 (40)	
	*H*41	8				2 (6.9)	6 (20.7)		
*strB*	Total	49	20 (37)	2 (16.7)	1 (33.3)	15 (51.7)	7 (24.1)	4 (80)	0.054
	*H*30Rx	30	20 (37)	1 (8.3)		8 (27.6)		1 (20)	
	*H*30R	3		1 (8.3)	1 (33.3)			1 (20)	
	*H*22	5				5 (17.2)			
	*H*27	2						2 (40)	
	*H*41	9				2 (6.9)	7 (24.1)		
Aminoglycoside and fluoroquinolone									
*aac*(*6'*)-*Ib*-*cr*	Total	55	39 (72.2)	6 (50)	0	3 (10.3)	7 (24.1)	0	**<0.001**
	*H*30Rx	53	39 (72.2)	5 (41.7)		3 (10.3)	6 (20.7)		
	*H*30R	2		1 (8.3)			1 (3.4)		
Beta-lactams									
*bla*_CTX-M-14_	Total	2	0	0	0	0	2 (6.9)	0	0.205
	*H*41	2					2 (6.9)		
*bla*_CTX-M-15_	Total	71	51 (94.4)	8 (66.7)	1 (33.3)	4 (13.8)	6 (20.7)	1 (20)	**<0.001**
	*H*30Rx	69	51 (94.4)	7 (58.3)	1 (33.3)	4 (13.8)	5 (17.2)	1 (20)	
	*H*30R	2		1 (8.3)			1 (3.4)		
*bla*_CTX-M-27_	Total	3	2 (3.7)	0	0	1 (3.4)	0	0	0.874
	*H*30Rx	2	2 (3.7)						
	*H*30R	1				1 (3.4)			
*bla*_CTX-M-55_	Total	4	0	3 (25)	1 (33.3)	0	0	0	**<0.001**
	*H*30Rx	1		1 (8.3)					
	*H*30R	3		2 (16.7)	1 (33.3)				
*bla*_OXA-1_	Total	54	38 (70.4)	7 (58.3)	0	3 (10.3)	6 (20.7)	0	**<0.001**
	*H*30Rx	52	38 (70.4)	6 (50)		3 (10.3)	5 (17.2)		
	*H*30R	2		1 (8.3)			1 (3.4)		
*bla*_TEM-1B_	Total	61	20 (37)	3 (25)	1 (33.3)	16 (55.2)	16 (55.2)	5 (100)	**0.036**
	*H*30Rx	33	20 (37)	1 (8.3)		6 (20.7)	4 (13.8)	2 (40)	
	*H*30R	13		2 (16.7)	1 (33.3)	8 (27.6)	1 (3.4)	1 (20)	
	*H*22	1					1 (3.4)		
	*H*27	2						2 (40)	
	*H*41	10				2 (6.9)	8 (27.6)		
*bla*_TEM-1C_	Total	3	0	0	0	0	3 (10.3)	0	0.05
	*H*22	3					3 (10.3)		
*bla*_KPC-3_	Total	6	0	0	0	6 (20.7)	0	0	**<0.001**
	*H*30Rx	6				6 (20.7)			
Macrolide									
*mphA*	Total	69	46 (85.2)	5 (41.7)	0	10 (34.5)	8 (27.6)	0	**<0.001**
	*H*30Rx	56	46 (85.2)	3 (25)		5 (17.2)	2 (6.9)		
	*H*30R	7		2 (16.7)		4 (13.8)	1 (3.4)		
	*H*41	4				1 (3.4)	3 (10.3)		
Chloramphenicol									
*catA1*	Total	4	0	0	0	0	2 (6.9)	2 (40)	**<0.001**
	*H*30Rx	1						1 (20)	
	*H*27	1						1 (20)	
	*H*41	2					2 (6.9)		
*catB3*	Total	51	37 (68.5)	6 (50)	0	2 (6.9)	6 (20.7)	0	**<0.001**
	*H*30Rx	48	37 (68.5)	5 (41.7)		1 (3.4)	5 (17.2)		
	*H*30R	3		1 (8.3)		1 (3.4)	1 (3.4)		
Sulfonamide									
*sul1*	Total	82	39 (72.2)	6 (50)	0	22 (75.9)	13 (44.8)	2 (40)	**0.01**
	*H*30Rx	60	39 (72.2)	4 (33.3)		13 (44.8)	3 (10.3)	1 (20)	
	*H*30R	11		2 (16.7)		7 (24.1)	2 (6.9)		
	*H*27	1						1 (20)	
	*H*41	8				2 (6.9)	6 (20.7)		
*sul2*	Total	52	20 (37)	2 (16.7)	1 (33.3)	18 (62.1)	8 (27.6)	3 (60)	**0.041**
	*H*30Rx	32	20 (37)	1 (8.3)		9 (31)	1 (3.4)	1 (20)	
	*H*30R	9		1 (8.3)	1 (33.3)	7 (24.1)			
	*H*27	2						2 (40)	
	*H*41	9				2 (6.9)	7 (24.1)		
Trimethoprim									
*dfrA12*	Total	8	8 (14.8)	0	0	0	0	0	**0.031**
	*H*30Rx	8	8 (14.8)						
*dfrA17*	Total	67	31 (57.4)	6 (50)	0	20 (69)	9 (31)	1 (20)	**0.014**
	*H*30Rx	50	31 (57.4)	4 (33.3)		12 (41.3)	2 (6.9)	1 (20)	
	*H*30R	9		2 (16.7)		6 (20.7)	1 (3.4)		
	*H*41	6				2 (6.9)	4 (13.8)		
*dfrA1*	Total	5	0	0	0	1 (3.4)	4 (13.8)	0	0.054
	*H*30Rx	1					1 (3.4)		
	*H*30R	1				1 (3.4)			
	*H*22	1					1 (3.4)		
	*H*41	2					2 (6.9)		
Tetracycline									
*tet*(A)	Total	86	40 (74.1)	8 (66.7)	1 (33.3)	18 (62.1)	15 (51.7)	4 (80)	0.292
	*H*30Rx	61	40 (74.1)	7 (58.3)		11 (37.9)	2 (6.9)	1 (20)	
	*H*30R	10		1 (8.3)	1 (33.3)	5 (17.2)	2 (6.9)	1 (20)	
	*H*22	4					4 (13.8)		
	*H*27	2						2 (40)	
	*H*41	9				2 (6.9)	7 (24.1)		

a Includes one publicly available sequence of *E. coli* ST131.

b Publicly available sequence data for *E. coli* ST131.

c Three isolates from Europe were from Lebanon.

d The *P* values are comparing the prevalence of each antibiotic resistance gene among geographical regions. *P* values in boldface type represent statistically significant results.

The aforementioned study from Japan (9) revealed higher prevalence of *bla*_CTX-M-14_ and *bla*_CTX-M-27_ in addition to *bla*_CTX-M-15_ among ESBL-*E. coli* isolates collected from another area in Japan. A study from Korea on *E. coli* isolates collected from 2012 to 2013 suggested that the majority of *H*30Rx isolates harbored *bla*_CTX-M-15_, whereas about half of the *H*30 non-Rx isolates harbored *bla*_CTX-M-14_ or *bla*_CTX-M-27_ (11). Geographical differences and different times of these studies may account for these discrepancies.

### Virotypes and virulence-associated genes.

The distribution of virotypes and virulence-associated genes among the *E. coli* ST131 strains is summarized in Table 3. The predominant virotypes differed across the geographical regions. The majority of *E. coli* ST131 isolates from the United States and half of those from Japan belonged to virotype C, whereas almost half of the isolates from Nepal belonged to virotypes other than A, B, C, D, and E. Two-thirds of *E. coli* ST131 isolates from Europe belonged to either virotype C or D. The virulence-associated genes such as *iha*, *sat*, *fyuA*, *traT*, *ompT*, and *malT* were highly prevalent among isolates from most geographical regions. The *papGII* gene was frequently observed only among isolates from Nepal and Tanzania, and *hlyA* and *cnf1* were common only in *E. coli* ST131 isolates obtained from Africa. Most of the virotypes obtained in our study belonged to virotype C (Table 1). Our finding that virotype C is the most prevalent virotype among *E. coli* ST131 isolates is consistent with previous reports on virotype distribution in ESBL-*E. coli* ST131 (4, 12). As previously suggested (4, 12), our results also suggested the association between virotype C and *H*30Rx sublineage. In our study, more isolates were identified as “other” virotype than previous studies (4, 12), which might be due to the different methodology used (e.g., WGS versus PCR). The majority of the ESBL-*E. coli* ST131 isolates (*n* = 28 [52%]) from Nepal were positive for *papGII* and *sat* but were negative for *hlyA*, and thus, were not categorized into any of the previously described virotypes (A to E) (13).

**TABLE 3:** Comparison of virotypes and virulence-associated traits and genes in *Escherichia coli* ST131

Virotype or virulence-associated trait and gene	*fimH* allele or sublineage	Total no. of isolates (*n* = 132)	No. of isolates (%) from:						*P* value^d^
			Nepal (*n* = 54)	Japan (*n* = 12)^a^	Other Asian countries (*n* = 3)^b^	USA (*n* = 29)^b^	Europe (*n* = 29)^b^^,^^c^	Africa (*n* = 5)^b^	
Virotypes									
A	Total	12	5 (9.3)	2 (16.7)	0	1 (3.4)	4 (13.8)	0	0.619
	*H*30Rx	9	5 (9.3)	1 (8.3)		1 (3.4)	2 (6.9)		
	*H*30R	1		1 (8.3)					
	*H*41	2					2 (6.9)		
B	Total	4	0	0	0	0	4 (13.8)	0	**0.012**
	*H*30Rx	1	0				1 (3.4)		
	*H*30R	1					1 (3.4)		
	*H*41	2					2 (6.9)		
C	Total	54	13 (24.1)	6 (50)	2 (66.7)	23 (79.3)	9 (31)	1 (20)	**<0.001**
	*H*30Rx	33	13 (24.1)	5 (41.7)	1 (33.3)	12 (41.4)	2 (6.9)		
	*H*30R	14		1 (8.3)	1 (33.3)	9 (31)	2 (6.9)	1 (20)	
	*H*41	4				2 (6.9)	2 (6.9)		
D^e^	Total	9	0	0	0	0	9 (31)	0	**<0.001**
	*H*22	9					9 (31)		
E	Total	18	10 (18.5)	3 (25)	0	2 (6.9)	1 (3.4)	2 (40)	0.09
	*H*30Rx	17	10 (18.5)	2 (16.7)		2 (6.9)	1 (3.4)	2 (40)	
	*H*30R	1		1 (8.3)					
Other	Total	35	26 (48.1)	1 (8.3)	1 (33.3)	3 (10.3)	2 (6.9)	2 (40)	**<0.001**
	*H*30Rx	28	26 (48.1)		1 (33.3)	1 (3.4)			
	*H*30R	2				2 (6.9)			
	*H*27	2						2 (40)	
	*H*41	3		1 (8.3)			2 (6.9)		
Virulence-associated traits and genes									
Adhesin									
* papGII*	Total	46	35 (64.8)	3 (25)	1 (33.3)	2 (6.9)	1 (3.4)	4 (80)	**<0.001**
	*H*30Rx	43	35 (64.8)	2 (16.7)	1 (33.3)	2 (6.9)	1 (3.4)	2 (40)	
	*H*30R	1		1 (8.3)					
	*H*27	2						2 (40)	
* iha*	Total	119	53 (98.1)	10 (83.3)	3 (100)	27 (93.1)	24 (82.8)	2 (40)	**0.001**
	*H*30Rx	86	53 (98.1)	8 (66.7)	2 (66.7)	16 (55.2)	6 (20.7)	1 (20)	
	*H*30R	16		2 (16.7)	1 (33.3)	9 (31)	3 (10.3)	1 (20)	
	*H*22	4					4 (13.8)		
	*H*41	10				2 (6.9)	8 (27.6)		
* hra*	Total	33	11 (20.4)	3 (25)	0	8 (27.6)	0	1 (20)	0.085
	*H*30Rx	11		3 (25)		8 (27.6)			
	*H*27	1						1 (20)	
Toxin									
* hlyA*	Total	20	10 (18.5)	3 (25)	0	2 (6.9)	1 (3.4)	4 (80)	**<0.001**
	*H*30Rx	7		2 (16.7)		2 (6.9)	1 (3.4)	2 (40)	
	*H*30R	1		1 (8.3)					
	*H*27	2						2 (40)	
* cnf1*	Total	19	9 (16.7)	3 (25)	0	2 (6.9)	1 (3.4)	4 (80)	**<0.001**
	*H*30Rx	7		2 (16.7)		2 (6.9)	1 (3.4)	2 (40)	
	*H*30R	1		1 (8.3)					
	*H*27							2 (40)	
* sat*	Total	118	54 (100)	10 (83.3)	3 (100)	26 (89.7)	22 (75.9)	3 (60)	**0.004**
	*H*30Rx	33		8 (66.7)	2 (66.7)	15 (51.7)	6 (20.7)	2 (40)	
	*H*30R	16		2 (16.7)	1 (33.3)	9 (31)	3 (10.3)	1 (20)	
	*H*22	4					4 (13.8)		
	*H*41	8				2 (6.9)	6 (20.7)		
Siderophore									
* iroN*	Total	11	0	0	0	0	11 (37.9)	0	**<0.001**
	*H*30Rx	1					1 (3.4)		
	*H*30R	1					1 (3.4)		
	*H*22	7					7 (24.1)		
	*H*41	2					2 (6.9)		
* fyuA*	Total	130	53 (98.1)	12 (100)	3 (100)	29 (100)	28 (96.6)	5 (100)	0.911
	*H*30Rx	87	53 (98.1)	8 (66.7)	2 (66.7)	16 (55.2)	6 (20.7)	2 (40)	
	*H*30R	19		3 (25)	1 (33.3)	11 (37.9)	3 (10.3)	1 (20)	
	*H*22	9					9 (31)		
	*H*27	2						2 (40)	
	*H*41	11		1 (8.3)		2 (6.9)	8 (27.6)		
* ireA*	Total	3	2 (3.7)	0	1 (33.3)	0	0	0	**0.009**
	*H*30Rx	3	2 (3.7)		1 (33.3)				
Protectins									
* kfiA*	Total	43	13 (23.6)	4 (33.3)	0	13 (44.8)	9 (31)	4 (80)	0.066
	*H*30Rx	28	13 (23.6)	2 (16.7)		9 (31)	2 (6.9)	2 (40)	
	*H*30R	8		2 (16.7)		4 (13.8)	1 (3.4)	1 (20)	
	*H*22	4					4 (13.8)		
	*H*27	1						1 (20)	
* iss*	Total	9	0	0	1 (33.3)	0	8 (27.6)	0	**<0.001**
	*H*30Rx	1			1 (33.3)				
	*H*30R	1					1 (3.4)		
	*H*22	5					5 (17.2)		
	*H*41	2					2 (6.9)		
Invasin									
* ibeA*	Total	9	0	0	0	0	9 (31)	0	**<0.001**
	*H*22	9					9 (31)		
Miscellaneous									
* traT*	Total	107	47 (87)	10 (83.3)	1 (33.3)	17 (58.6)	29 (100)	3 (60)	**<0.001**
	*H*30Rx	68	47 (87)	7 (58.3)		6 (20.7)	6 (20.7)	2 (40)	
	*H*30R	16		3 (25)	1 (33.3)	9 (31)	3 (10.3)		
	*H*22	9					9 (31)		
	*H*27	1						1 (20)	
	*H*41	10				2 (6.9)	8 (27.6)		
* ompT*	Total	129	54 (100)	12 (100)	2 (66.7)	28 (96.6)	28 (96.6)	5 (100)	**0.01**
	*H*30Rx	87	54 (100)	8 (66.7)	1 (33.3)	16 (55.2)	6 (20.7)	2 (40)	
	*H*30R	18		3 (25)	1 (33.3)	10 (34.5)	3 (10.3)	1 (20)	
	*H*22	9					9 (31)		
	*H*27	2						2 (40)	
	*H*41	11		1 (8.3)		2 (6.9)	8 (27.6)		
* malX*	Total	131	54 (100)	12 (100)	3 (100)	29 (100)	28 (96.6)	5 (100)	0.911
	*H*30Rx	88	54 (100)	8 (66.7)	2 (66.7)	16 (55.2)	6 (20.7)	2 (40)	
	*H*30R	19		3 (25)	1 (33.3)	11 (37.9)	3 (10.3)	1 (20)	
	*H*22	9					9 (31)		
	*H*27	2						2 (40)	
	*H*41	11		1 (8.3)		2	8 (27.6)		

a Includes one publicly available sequence of *E. coli* ST131.

b Publicly available sequence data for *E*. *coli* ST131.

c Three isolates from Europe were from Lebanon.

d The *P* values are comparing the prevalence of each antibiotic resistance gene among geographical regions. *P* values in boldface type represent statistically significant results.

e All isolates of virotype D were identified as D3.

### **Plasmid replicon types**.

The distribution of plasmid replicon types was similar across geographical regions except that FIA was prevalent in Nepal and the United States and considerably less prevalent in Europe; other geographical regions showed intermediate prevalence (Table 4). FII was commonly found among ESBL-*E. coli* ST131 isolates from Nepal, whereas it was considerably less prevalent in Japan. IncP was detected in 40% of *E. coli* ST131 isolates from Africa, but it was very rarely found or not found in isolates from other areas.

**TABLE 4:** Comparison of plasmid replicon types of *Escherichia coli* ST131

Replicon type	*fimH* allele or sublineage	Total no. of isolates	No. of isolates (%) from:						*P* value^d^
			Nepal (*n* = 54)	Japan (*n* = 12)^a^	Other Asian countries (*n* = 3)^b^	USA (*n* = 29)^b^	Europe (*n* = 29)^b^^,^^c^	Africa (*n* = 5)^b^	
IncFIA	Total	84	38 (70.4)	6 (50)	2 (66.7)	25 (86.2)	10 (34.5)	3 (60)	**0.002**
	*H*30Rx	62	38 (70.4)	4 (33.3)	1 (33.3)	14 (48.3)	3 (10.3)	2 (40)	
	*H*30R	17		2 (16.7)	1 (33.3)	11 (37.9)	2 (6.9)	1 (20)	
	*H*41	2					2 (6.9)		
IncFIB	Total	89	40 (74.1)	8 (66.7)	1 (33.3)	14 (48.3)	23 (79.3)	3 (60)	0.09
	*H*30Rx	48	40 (74.1)	5 (41.7)		2 (6.9)	1 (3.4)		
	*H*30R	18		3 (25)	1 (33.3)	10 (34.5)	3 (10.3)	1 (20)	
	*H*22	9					9 (31)		
	*H*27	2						2 (40)	
	*H*41	10				2 (6.9)	8 (27.6)		
IncFIC	Total	3	0	0	0	0	3 (10.3)	0	0.053
	*H*22	1					1 (3.4)		
	*H*41	2					2 (6.9)		
IncFII	Total	95	43 (79.6)	4 (33.3)	1 (33.3)	23 (79.3)	21 (72.4)	3 (60)	**0.017**
	*H*30Rx	60	43 (79.6)	2 (16.7)		11 (37.9)	2 (6.9)	2 (40)	
	*H*30R	17		2 (16.7)	1 (33.3)	10 (34.5)	3 (10.3)	1 (20)	
	*H*22	8					8 (27.6)		
	*H*41	8				2 (6.9)	6 (20.7)		
IncI1	Total	9	3 (5.6)	2 (16.7)	1 (33.3)	1 (3.4)	2 (6.9)	0	0.29
	*H*30Rx	5	3 (5.6)	1 (8.3)		1 (3.4)			
	*H*30R	2		1 (8.3)	1 (33.3)				
	*H*41	2					2 (6.9)		
IncFrepB	Total	40	13 (24.1)	3 (25)	0	15 (51.7)	7 (24.1)	2 (40)	0.091
	*H*30Rx	32	13 (24.1)	2 (16.7)		12 (41.4)	3 (10.3)	2 (40)	
	*H*30R	4		1 (8.3)		3 (10.3)			
	*H*22	4					4 (13.8)		
IncB/O	Total	1	0	0	0	1 (3.4)	0	0	0.611
	*H*30R	1				1 (3.4)			
IncY	Total	2	0	0	0	1 (3.4)	1 (3.4)	0	0.763
	*H*30Rx	1					1 (3.4)		
	*H*30R	1				1 (3.4)			
IncN	Total	8	0	2 (16.7)	0	2 (6.9)	4 (13.8)	0	0.092
	*H*30Rx	6		1 (8.3)		2 (6.9)	3 (10.3)		
	*H*30R	1		1 (8.3)					
	*H*22	1					1 (3.4)		
IncP	Total	3	0	0	0	0	1 (3.4)	2 (40)	**<0.001**
	*H*30R	1					1 (3.4)		
	*H*27	2						2 (40)	
IncA/C	Total	2	0	0	0	2 (6.9)	0	0	0.205
	*H*30R	2				2 (6.9)			

a Includes one publicly available sequence of *E. coli* ST131.

b Publicly available sequence data for *E. coli* ST131.

c Three isolates from Europe were from Lebanon.

d The *P* values are comparing the prevalence of each antibiotic resistance gene among geographical regions. *P* values in boldface type represent statistically significant results.

The association of *bla*_CTX-M-15_ and IncF replicon has been reported previously (14), and the *H*30Rx sublineage was created by introduction of IncF (15). We previously found that a plasmid resembling pEC958 and harboring FIA and FII (16) was present in approximately 80% of the ESBL-*E. coli* ST131 isolates from Nepal (5). The high prevalence of IncF (IncFIA, FIB, and FII) observed among ESBL-*E. coli* ST131 isolates from Nepal is consistent with these previous reports and is reasonable considering the high prevalence of *H*30Rx among ESBL-*E. coli* ST131 isolates.

### Phylogenetic analysis of *E. coli* ST131 in different geographical regions.

In the phylogenetic analysis based on WGS (Fig. 1), the majority of ESBL-*E. coli* ST131 isolates from Nepal clustered together, whereas those from Japan were more diverse. Thus, ESBL-*E. coli* ST131 may have been introduced into Japan more sporadically over time. Strict requirements for antibiotic prescription in Japan might have resulted in relatively low selective pressure, along with more advanced medical and social infrastructure. Carriers of ESBL-*E. coli* are usually placed under contact precaution in health care facilities in Japan, including the hospital in this study, to minimize transmission. These factors might explain at least in part the differences in the prevalence and clonality of ST131 between these two Asian countries. In contrast, prevalent clonal spread of ESBL-*E. coli* appears to have occurred in Nepal, where poor infection control practices and sanitation might facilitate dissemination of antimicrobial-resistant strains. In Nepal, antibiotics can be purchased in the community at general retail stores and pharmacies. According to recent reports, 80% of the drugs are purchased outside the government-supplied health system (17, 18). In addition, inappropriate prescription occurs in up to 40% of patients (17, 18).

*E. coli* ST131 isolates from the United States are distributed across the phylogeny, even those isolates that were collected in the same year. The wide diversity of population in the United States might explain in part this phylogenetic characteristic. *E. coli* ST131 isolates from Europe (including three *E. coli* ST131 isolates from Lebanon) could be roughly divided into four clusters, with one major cluster consisting mainly of *E. coli* ST131 isolates from Denmark and one cluster consisting of two *E. coli* ST131 isolates from Lebanon. *E. coli* ST131 isolates from Denmark predominantly consisted of *E. coli* ST131 from Europe (72%). Four *E. coli* ST131 isolates from Tanzania and one isolate from South Africa were included in *E. coli* ST131 from Africa. *E. coli* ST131 isolates from Africa were phylogenetically diverse. *E. coli* ST131 isolates from Asian countries other than Nepal and Japan included one isolate from South Korea, one isolate from India, and one isolate from Thailand. They did not belong to the same cluster.

There are several limitations to this study. Since ESBL-*E. coli* ST131 isolates from Nepal and Japan were collected during a relatively short period of time, epidemiologically related isolates may have been included in the analysis, and the trends across years could not be elucidated. However, as shown in Fig. 1, the distribution of ESBL-*E. coli* ST131 isolates in each region suggests that the contribution of such clonal isolates was unlikely to have been remarkable. Due to the small number of isolates from Japan, the power of the statistical comparisons between isolates from Nepal and Japan is limited. Also, only small numbers of *E. coli* ST131 isolates from some geographical regions such as Africa could be included due to limited publicly available data. Finally, the difference in the molecular analyses, i.e., WGS and PCR, used might have caused the difference in the identification of the target genes.

In conclusion, comparative analysis of ESBL-*E. coli* ST131 genomes from Japan and Nepal and those from other geographical regions revealed distinct phylogenetic characteristics of the spread of ESBL-*E. coli* ST131 in these two geographical areas of Asia. Multiple yet distinct factors might contribute to the local spread of ESBL-*E. coli* ST131 in each region.

## MATERIALS AND METHODS

### Isolates and susceptibility testing.

All ESBL-*E. coli* isolates were serially collected from the Tribhuvan University Teaching Hospital (444 beds), Kathmandu, Nepal, between 1 February 2013 and 31 July 2013 and from the National Center for Global Health and Medicine (NCGM) (781 beds), Tokyo, Japan, between 1 October 2013 and 30 September 2014; both centers are tertiary teaching hospitals. Some of the isolates from Nepal were included in our previous study (5).

The *E. coli* strains from both Nepal and Japan were identified, and their susceptibility was tested in accordance with the Clinical and Laboratory Standards Institute (CLSI) criteria (19) by using an automated broth microdilution system (MicroScan; Siemens AG, Germany) unless otherwise stated. The MIC of fosfomycin was determined using an NC6.11J panel (Siemens AG, Germany) which contains glucose-6-phosphate, and its susceptibility status was determined as previously reported (20). ESBL production was confirmed by disc diffusion tests in accordance with the 2009 CLSI criteria (19). If multiple ESBL-*E. coli* isolates were identified from a patient during the study period, only the first isolate was included.

### Whole-genome sequencing and phylogenetic analysis.

Molecular analysis was conducted at the Department of Infectious Diseases, Research Institute, National Center for Global Health and Medicine, Tokyo, Japan. The strains were cultured overnight in lysogeny broth (LB) (Nakarai Tesque, Kyoto, Japan), and genomic DNA was purified using a DNeasy blood and tissue kit (Qiagen, Venlo, Netherlands). The genomes of the isolates were then subjected to MiSeq sequencing by using Nextera XT library kits (Illumina, Inc., San Diego, CA), according to the manufacturer’s instructions. Approximately 1 million paired-end reads (301 bp × 2) were obtained from each genome and analyzed using the CLC Genomics Workbench software (CLC Bio, Aarhus, Denmark). The reads from each isolate were trimmed by screening for base quality (quality score limit of 0.05; reads that contained greater than two ambiguous nucleotides or that were less than 15 bp in length were removed) (21). The resulting sequencing data were registered with the DNA Data Bank of Japan (DDBJ) (DDBJ accession no. DRA003515). Velvet and Mummer (open source MUMmer 3.0) described previously were used for *de novo* assembly to prepare contigs and single nucleotide polymorphism (SNP) calling, respectively (21, 22).

All genome sequence data for ST131 strains available up to January 2015 were downloaded from the NCBI database (http://www.ncbi.nlm.nih.gov/genome/genomes/167?genome_assembly_id=group161531; accessed 26 September 2016) and included for phylogenetic analysis to determine the sequence diversity. SNP concatemers were prepared using a custom script and aligned using MAFFT (23). *E. coli* SE15 (accession no. NC_013654.1) was used as the reference to call SNPs of the isolates after mobile elements were removed from the chromosome. The appropriate evolutionary model (transversion model plus gamma distribution) was determined using jModelTest2 (24). Maximum likelihood (ML) phylogenetic trees were estimated using PHYML 3.0 (25). Branch support for nodes was assessed using the Shimodaira and Hasegawa (SH) test implemented in PHYML.

The contigs were subjected to further analyses by using the BLAST algorithm (26) and ResFinder (27) to identify whether virulence marker genes and drug resistance genes were present in the genomes (28, 29). An identification rate of more than 97% was considered positive for each target gene. Phylotypes (30), virulence genotypes (31), *fimH* alleles and *H*30R and *H*30Rx sublineages of ST131 (10), and distribution of acquired drug resistance genes were determined. The nucleotide sequences corresponding to the ST131 multilocus sequence typing (MLST) allelic profile (*adk53*, *fumC40*, *gyrB47*, *icd13*, *mdh36*, *purA28*, and *recA29*) were downloaded from the University of Warwick (http://mlst.warwick.ac.uk/mlst/) and compared by BLAST algorithm to the WGS data (3, 26). Virotypes were determined based on the virulence gene scheme (13). Sequence typing of plasmid replicons was performed as described elsewhere (32, 33).

We added one *E. coli* ST131 isolate from Japan for which sequence data were available publicly (http://www.ncbi.nlm.nih.gov/genome/genomes/167?genome_assembly_id=group161531; accessed 26 September 2016). SE15 is a completely sequenced ST131 representative strain, which was used as a reference in the current study and a previous study as well (3).

### Statistical analysis.

All statistical analyses were performed using IBM-SPSS statistics 20 (2012). Bivariate analyses were performed using Fisher’s exact test or chi-square test for categorical variables and the Mann-Whitney U test for continuous variables. All *P* values were two sided. The percentage values included in this article are the “valid percentages,” which exclude the missing data.

### Accession number(s).

Sequence data were deposited in the DNA Data Bank of Japan (DDBJ) under accession no. DRA003515.
