# Correction for Miyoshi-Akiyama et al., “Comparative Genome Analysis of Extended-Spectrum-β-Lactamase-Producing *Escherichia coli* Sequence Type 131 Strains from Nepal and Japan”

**DOI:** 10.1128/mSphere.00136-17

**Published:** 2017-05-24

**Authors:** Tohru Miyoshi-Akiyama, Jatan Bahadur Sherchan, Yohei Doi, Maki Nagamatsu, Jeevan B. Sherchand, Sarmila Tandukar, Norio Ohmagari, Teruo Kirikae, Hiroshi Ohara, Kayoko Hayakawa

**Affiliations:** a Pathogenic Microbe Laboratory, Research Institute, National Center for Global Health and Medicine, Tokyo, Japan; b Department of Clinical Microbiology, Kathmandu University School of Medical Sciences, Dhulikhel, Nepal; c Division of Infectious Diseases, University of Pittsburgh School of Medicine, Pittsburgh, Pennsylvania, USA; d Disease Control and Prevention Center, National Center for Global Health and Medicine, Tokyo, Japan; e Department of Infectious Diseases, Research Institute, National Center for Global Health and Medicine, Tokyo, Japan; f Public Health Research Laboratory, Institute of Medicine, Tribhuvan University Teaching Hospital, Kathmandu, Nepal; g Department of International Medical Cooperation, National Center for Global Health and Medicine, Tokyo, Japan

## AUTHOR CORRECTION

Volume 1, no. 5, e00289-16, 2016, https://doi.org/10.1128/mSphere.00289-16. Due to the classification definitions that we used for our work, six isolates were misclassified for the *fimH* allele or sublineage in our paper. The revised *fimH* alleles or sublineages are as follows: HVH186 (H30Rx), J069 (H30Rx), N439/N1027/N1471 (H30R [non-Rx]), and N1449 (H41). A revised figure and revised tables are available from the authors upon request. (We are extremely grateful to Yasufumi Matsumura, Kyoto University Graduate School of Medicine, Japan, for his tremendous help on molecular analysis.)

